# Hematometrocolpos Due to an Imperforate Hymen

**DOI:** 10.5334/jbr-btr.1413

**Published:** 2018-01-03

**Authors:** Thomas Bekaert, Kristof Ramboer

**Affiliations:** 1 University Hospital Leuven, BE; 2 AZ St.-Lucas, Bruges, BE

**Keywords:** Hematometrocolpos, Hymen, Müllerian duct abnormality

A 14-year-old girl presented to the emergency department. She complained of chronic back pain that recently started to irradiate towards the abdomen. The pain was persistent and progressive. The blood analysis showed a slight increase in white blood cell count and C-reactive protein (CRP).

Subsequently abdominal ultrasound (US) was requested. A large well-circumscribed hypogastric mass was seen. The mass had a hyporeflective content and a small amount of hyperreflective material posteriorly, compatible with debris in a midline cyst (Figure 1).

**Figure 1:**
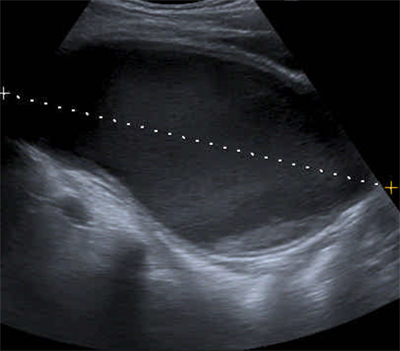
Sagittal US-image showing a large hypogastric mass with hyporeflective content and a small amount of hyperreflective material posteriorly.

The day after, magnetic resonance imaging (MRI) was performed. The mass appeared to be a dilatation of the vagina, in continuity with the uterine cavity (Figure 2A, sagittal image: arrow = uterine cervix). The content was T2 hyper- to iso-intense and T1 hyperintense (Figure 2B, axial image). The hyperreflective material seen on ultrasound, showed susceptibility artifacts on T2*-weighted images, which corresponds to hemosiderin (Figure 2C, axial image). There was no pathologic diffusion restriction.

**Figure 2:**
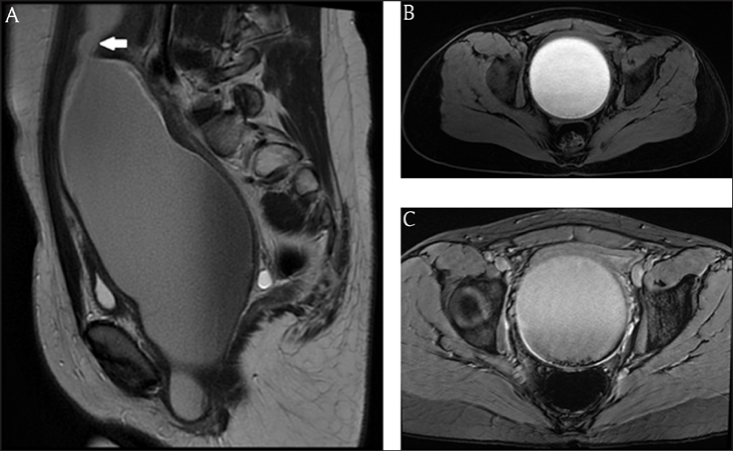
MRI: **A.** Sagittal T2-weighted image showing a large cystic mass with T2 hyper- to iso-intense content. Arrow = uterine cervix. **B.** Axial T1-weighted image showing a large cystic mass with T1 hyperintense content. **C.** Axial T2*-weighted image showing susceptibility artifacts posteriorly in the lesion.

After MRI, the patient was seen by a gynecologist. He found out that the abdominal pain had a cyclic pattern on a monthly base, and that the patient never had menses. On clinical examination, a mass was felt and the hymen was bombed and painful.

Based on these findings, the diagnosis of a hematometrocolpos, due to an imperforate hymen, was made. The patient underwent hymenotomy to remove the large amount of blood.

## Comment

An imperforate hymen is typically not identified until menarche, when it manifests in association with cyclic pelvic pain and primary amenorrhea; imaging may reveal a hematometrocolpos. The same characteristics can be seen with several Müllerian duct anomalies (MDA), such as a low transverse vaginal septum. In contradiction to a vaginal septum, an imperforate hymen is not a real MDA but just a pathological persistent closure of the membrane. It is imperative that the renal tract is investigated for congenital anomalies when an MDA is suspected, but this is not necessary in patients with hemato(metro)colpos due to an imperforate hymen, since this pathology usually appears as an isolated finding.

US is the initial imaging modality in these young patients. Once the possibility of an MDA is raised, MRI is recommended [1].
